# Revisiting Provider Communication to Support Team Cohesiveness: Implications for Practice, Provider Burnout, and Technology Application in Primary Care Settings

**DOI:** 10.1155/2022/9236681

**Published:** 2022-06-07

**Authors:** Allison A. Norful, Yun He, Adam Rosenfeld, Cilgy M. Abraham, Bernard Chang

**Affiliations:** ^1^Columbia University School of Nursing, 630 West 168^th^ Street- Mail Code 6, New York, NY 10032, USA; ^2^Columbia University Mailman School of Public Health, Department of Biostatistics, New York, NY, USA; ^3^Columbia University Mailman School of Public Health, Department of Sociomedical Science, New York, NY, USA; ^4^Columbia University School of Nursing, New York, NY, USA; ^5^Columbia University Irving Medical Center, Department of Emergency Medicine, New York, NY, USA

## Abstract

**Background:**

Effective team communication is an essential aspect of care delivery and the coordination of patients in primary care settings. With the rapid evolution of health information technology (HIT), including the implementation of electronic health records, there remains a gap in the literature about preferred methods of primary care team communication and the subsequent impact of provider and team outcomes (e.g., team cohesiveness; burnout). This study explores the impact of varying modes of communication across provider disciplines and by geographic settings during primary care delivery.

**Methods:**

We used a cross-sectional survey design to collect data from a random convenience sample of PCPs (physicians, nurse practitioners, and physician assistants) (*n* = 314) in New York State (NYS). We mailed a paper survey with validated measures for communication methods, team cohesiveness, and provider outcomes (burnout, job dissatisfaction, and the intention to leave position). Descriptive statistics, linear regression models, and crude and adjusted odds ratios while controlling for individual and practice characteristics were calculated.

**Results:**

In-person communication was found to yield greater job satisfaction and less intention to leave current position in the next year (*p*=0.02) compared to other forms of communication including electronic health record features. The odds of job satisfaction was 1.51 times higher with in-person communication (OR: 1.51, 95% CI: 1.05, 2.19), and the odds of intending to leave a position was 45% less with in-person communication (OR: 0.55, 95% CI: 0.36, 0.85). The odds of reporting burnout at work was 36% less with in-person communication (OR: 0.64, 95% CI: 0.43, 0.92) compared to other communication modalities. There was no significant association between team communication via the EHR and team cohesiveness, provider burnout, or job satisfaction.

**Conclusion:**

This study demonstrates evidence that in-person communication is more likely to reduce burnout and job dissatisfaction compared to other forms of communication infrastructure in primary care settings. More research is needed to understand PCP perspectives about the functionality and potential burden that inhibits the use of EHR features for provider-provider communication. In addition, attention to the needs of teams by geographic location and by workforce discipline is warranted to ensure effective HIT communication application adoption.

## 1. Introduction

A number of recent developments in the healthcare landscape have put the primary care system in the United States under immense stress. An aging population with complex care requirements combined with physician shortages has exacerbated the strain on the healthcare workforce to meet all demands for care [1]. One study of primary care providers found that the hours necessary to deliver high-quality care for patients with chronic conditions exceeded the number of hours physicians were actually available [2]. This increased demand has prompted calls for systematic improvements in primary care delivery to support complex coordination of patient care responsibilities and alleviate provider strain.

Exploration of team-based collaboration to increase quality and continuity of care offers an innovative route to meet primary care delivery demand despite threatened workforce supply shortages [3]. An increased amount of evidence over the past decade supports the use of interdisciplinary teams in outpatient settings [4, 5]. In fact, effective team-based care has been linked to better clinical patient outcomes, decreased hospitalizations, increased patient safety and access to care, and improved coordination of patients across care transitions [6–9]. Isolating attributes of team-based care (e.g., poor provider communication) that may inhibit optimal outcomes is critical to implement effective team models across clinical settings [10].

Information transfer between clinical team members (e.g., communication strategies) is a foundational attribute in patient care delivery and is needed to effectively manage a patient's coordination of services [11, 12]. The consequences of poor team communication on patient health and safety are well documented. In a study of family physician error reports, team miscommunication was found to have initiated the majority of errors, of which many resulted in patient harm [13]. Another study, conducted though the observation of surgical teams, found that a third of communication errors led to poor procedural outcomes including patient safety risk [14]. On the contrary, improved interdisciplinary communication between clinicians has been linked to optimal patient outcomes. For example, one study found that improvements in nurse-physician communication and collaboration significantly decreased healthcare-associated infections [15]. Despite an extensive body of literature about team communication in hospital-based and acute care settings [16, 17], evidence about communication practices among primary care teams is lagging behind.

In response to financial incentives through Medicare and Medicaid, the current landscape of primary care settings has seen an exponential increase in the adoption of HIT, specifically electronic health record (EHR) infrastructure. Some systems are equipped with a secure messaging feature used for communication between patients and providers or clinical team members [16]. However, evidence about the effectiveness of HIT for clinical team communication in primary care, including provider-provider communication preferences or perceived burden, is substantially limited compared to acute care settings [18]. Further, it remains unclear if the increased use of HIT for team communication in primary care yields harmful burnout or job dissatisfaction outcomes. Most research about the association between HIT and provider burnout has focused on documentation burden, and EHR functionality or usability [19]. There is limited evidence about how providers use EHRs for team communication in primary care and the potential implications for burnout and job satisfaction outcomes.

To add to the complexity of primary care team communication, it is also important to consider that there are varying perspectives across clinical disciplines about effective strategies to improve communication. In a meta-analysis of studies measuring attitudes toward effective interdisciplinary collaboration strategies, including communication, researchers found differing perceptions between nurses and physicians [20], thereby suggesting that communication infrastructure preferences may be discipline-specific. The use of HIT for team communication may also vary across geographic settings given noted evidence of decreased EHR adoption in rural-based primary care settings [21]. Researchers and policy makers are calling for a closer examination of communication preferences, including the expanded use of HIT to understand and inform proactive communication strategies for team communication that alleviate provider burden including burnout [22]. A current and targeted investigation of interdisciplinary primary care team communication modalities, HIT infrastructure, and impact on provider outcomes across varying types of primary care settings is warranted. The aims of this study are to (1) describe current communication strategies in primary care settings across geographic settings and by workforce disciplines; (2) investigate what types of provider communication infrastructure is supportive of optimal team cohesiveness; and (3) determine which type of communication modalities reduce adverse provider outcomes (burnout, job dissatisfaction, and intention to leave current position) in primary care settings. We hypothesize that increased use of electronic forms of communication (e.g., secure messaging in the electronic health record) will be positively associated with burnout, job dissatisfaction, and the intention to leave. Further, we also hypothesize that in-person communication will increase the odds of team cohesiveness.

## 2. Materials and Methods

### 2.1. Data Collection

Details from the parent study can be found elsewhere [23]. We used a cross-sectional survey design to collect data from a random sample of PCPs across New York State (NYS) recruited from the IQVIA (formerly SKA) database [24]. IQVIA contains nearly the entire population of ambulatory-based providers and is updated every 6 months. Currently, physicians, nurse practitioners (NPs), and physician assistants (PAs) deliver the majority of primary care services in the country [25]. IQVIA database includes up-to-date information on physicians', NPs', and PAs' names, practice locations, network affiliations, email addresses, and National Provider Identifier numbers. This data source has an advantage over other sources in that it also contains information about patient volume, number of providers, site specialty, and ownership that are not available elsewhere [26]. We identified primary care practices in NYS with at least 2 PCPs to adequately investigate team cohesiveness among interdisciplinary PCP disciplines. NYS was selected due to its close proximity to the researcher, known target sample size, and budgetary considerations [27]. NPs were eligible if their practice location and certification was in one of the specializations: adult, family, gerontology, or primary care [28]. PAs were eligible if they practiced in a primary care outpatient setting, and physicians were eligible if they were identified as internists, family physicians, or geriatricians [29].

Ethical approval was obtained from the Columbia University Irving Medical Center institutional review board. We performed a power analysis and determined a sample size of 267 PCPs (physicians, NPs, and PAs) had a power of 0.8 to reject the null hypothesis with a Type I error of 0.05. We mailed a paper survey to participants using their practice addresses obtained from IQVIA. A letter and a consent form were included in the survey, describing the purpose of the study, its voluntary nature, and the confidentiality of responses. Our use of a mail survey was driven in part by past work on mail surveys of healthcare providers finding such approaches to yield the highest response rate [30]. We asked the participants to complete the paper survey and return it in an enclosed prepaid envelope to the research team. The estimated completion time of each survey was 10–15 minutes. Each survey had an identification number assigned to it for respondent tracking and survey completion. Using the *Dillman* method for mailed surveys, nonrespondents received a reminder postcard approximately 3 weeks after the first mailing [31]. After an additional 3 weeks, we conducted a second mailing for nonrespondents. Participants were offered an opportunity to participate in a lottery drawing for one of thirty *fitbit* wireless activity trackers. Incentives in workforce studies have been found to improve return rates [32, 33].

### 2.2. Survey Measures

Individual PCP demographics, practice characteristics, and practice structural capabilities were collected at the beginning of each survey. PCP demographics included age, gender, race, job title, education, work experience, and certification type. Practice characteristics included geographic setting (e.g., urban), practice size (#PCPs), patient volume, practice ownership, patient panel size, and average number of hours worked per week. Practice structural capabilities included electronic health record resources and reported proximity of each comanaging provider (e.g., “Do you work in the same practice location as your comanaging provider?”).

Team cohesiveness was measured using the *Provider CoManagement Index*, a 20-item instrument that asks providers to rate the degree to which comanagement characteristics are present in their practices. For example, “My comanaging provider and I communicate patient needs in a timely manner.” A 4-point Likert response scale from *“strongly agree” (4) to “strongly disagree” (1)* is used. The tool and its 3 subscales have high internal consistency reliability: Effective Communication (*α* = 0.811); Mutual Respect and Trust (*α* = 0.746); and Shared Philosophy of Care (*α* = 0.779). Higher mean scores on each subscale indicate better provider relations. A mean score for each subscale and the overall scale was computed.

Burnout, job satisfaction, and intention to leave current position was measured using 3 validated items used in large scale survey research supported by the Agency of Health Research Quality (AHRQ) and Health Resources and Services Administration (HRSA). The first item “What is your overall level of satisfaction with your principal position?” used a 4-point Likert response category ranging from “Very Satisfied” to “Very Dissatisfied.” Workforce retention was measured using the item, “Do you plan to leave your principal position?” (Response categories: “Yes plan to leave in 2018,” “Yes, plan to leave in 1-2 years,” “No plans to leave in next 2 years,” and “Undecided”). Both of these items have been used on well-validated tools, such as the National Sample Survey of Nurse Practitioners [34]. Burnout was measured using an item from the AHRQ public domain (*Mini-Z burnout study*) [35] and has been used in multiple national workforce studies to assess provider burnout. There are 5 response options that range from “I enjoy my work. I have no symptoms of burnout” to “I feel completely burned out and often wonder if I can go on.”

### 2.3. Statistical Analysis

The survey data was manually entered into a spreadsheet, cleaned and coded, and exported to R statistical software [36] for data analysis. Normality of continuous variables was checked through the Shapiro–Wilk tests. Sample characteristics, including demographics, practice characteristics, smartphone use, and modes of communication, were analyzed and presented as medians and interquartile ranges (IQR) for continuous variables and absolute and relative frequencies (%) for categorical variables. Differences between locations were evaluated using the Kruskal–Wallis tests for continuous variables and chi-squared tests or Fisher's exact tests for categorical variables. The frequency of using different modes of communication was measured as four levels, including Frequently, Often, Rarely, and Never. Spearman's correlation was used to assess the correlation between the total PCMI score and different modes of communication. For example, chi-squared tests or Fisher's exact tests were used to assess the correlation between burnout-related outcomes and different modes of communication. To control for confounders like demographics and practice characteristics, logistic regression models were built to further assess the correlation between PCMI/burnout-related outcomes and different modes of communication. In logistic regression models, the total PCMI score was dichotomized using the mean score. *p* values were attained from Wald tests. Two-sided statistical tests were performed with the significance level set at *p* ≤ 0.05 using the R software package (v. 3.6.2).

## 3. Results

In total, 314 eligible participants were analyzed after removing missing data. The sample included 110 (35.0%) PCPs working in urban areas, 70 (22.3%) in rural areas, and the remainder in suburban areas. There was no significant difference of discipline distribution by location (*p*=0.96). The demographics and practice characteristics of the study population are shown in Table 1 by their primary geographic location. The median age overall was 55 years with IQR (44, 62). The median years of experience overall was 20 years with IQR (12, 29). The sample of participants was predominantly White (*n* = 270; 86.3%) and female (*n* = 237; 75.7%). Most of the urban or suburban providers worked in physician-owned practices, while most of the rural providers worked in a university/hospital affiliated clinic. Over half (57.2%) of respondents had 10 years or more of experience in their field and 183 (58.3%) worked part-time. Most urban or suburban providers comanaged/shared a panel of patients with other physicians or providers, while most of the rural providers independently managed an independent panel of patients.

### 3.1. Smartphone Use and Communication Mode

Although the prevalence of using a smartphone when managing patient care and the mode of communication providers used when discussing patient care with another provider were not significantly different between physicians, Pas, and NPs (see Table 1), differences were found between their primary practice locations (see Table 2). Significantly fewer providers in rural communities reported using a smartphone compared to PCPs in urban and suburban settings. No significant difference was found for smartphone use reasons (e.g., clinical decision support; electronic prescribing) by practice location. The prevalence of using in-person communication, telephone call, text message, and email to discuss patient care with another provider was significantly different between locations (see Table 2). Among all participants, in-person communication was mostly used, while smartphone applications were least used. Among providers practicing in rural areas, 38 PCPs (54.3%) communicated via in-person exchanges frequently, 9 (13.2%) used telephone calls frequently, and 3 (4.6%) sent emails for communication frequently. No providers practicing in rural areas reported using text messages for communication. Less than half (48.7%) of all providers used Electronic Health Records for provider-provider communication frequently. Only 12 (4.3%) of all providers used smartphone applications for communication. The providers practicing in rural areas were significantly less likely to use the EHR or smartphones to discuss patient care with another provider compared to providers in urban or suburban areas.

### 3.2. Correlation between Modes of Communication and Provider-Related Outcomes

Bivariate correlations between modes of communication and provider outcomes (e.g., team cohesiveness, burnout, job satisfaction) are shown in Table 3. Team cohesiveness, measured by the total PCMI score, was found to be positively associated with in-person communication, telephone calls, and text message. The more frequently in-person communication, telephone call, or text message was used, the higher the total PCMI score (Spearman's correlation coefficient: in-person communication: 0.29, *p* < 0.001; telephone call: 0.15, *p*=0.008; text message: 0.15, *p*=0.009). In-person communication was also found to be associated with increased job satisfaction (*p*=0.02) and a decreased intention to leave the current position in the next year (*p*=0.02).

Correlations between modes of communication and team/provider-related outcomes after controlling for occupation, age, race, gender, years of experience, office setting, length of work experience, work type and location are displayed in Table 4. The frequency of modes of communication by discipline is displayed in Table 5. After adjustment, in-person communication, telephone call, and text message remained associated with the total PCMI score. The odds of self-reported effective team cohesiveness were 2.53 times higher with one unit increase in the frequency of using in-person communication (OR: 2.53, 95% CI: 1.7, 3.86). Similarly, the odds of self-reported effective team cohesiveness were approximately 1.40 times higher with one unit increase in the frequency of using telephone call or text message (OR of using telephone call: 1.41, 95% CI: 1.03, 1.93; OR of using text message: 1.38, 95% CI: 1.03, 1.86). In addition, in-person communication remained associated with job satisfaction and the intention to leave current position. The odds of feeling satisfied with the current job were 1.51 times higher with one unit increase in the frequency of using in-person communication (OR: 1.51, 95% CI: 1.05, 2.19). The odds of intending to leave current position were 45% less with one unit increase in the frequency of using in-person communication (OR: 0.55, 95% CI: 0.36, 0.85). Moreover, after controlling for individual and practice characteristics, in-person communication was found to be significantly associated with decreased burnout. The odds of reporting burnout at work were 36% less with one unit increase in the frequency of using in-person communication (OR: 0.64, 95% CI: 0.43, 0.92).

## 4. Discussion

This study presents current evidence about the types of communication modalities used by PCPs across geographic settings and workforce disciplines and their subsequent impact on team and provider-level outcomes. In-person communication was found to have a significant impact on increasing job satisfaction as well as improving team relations. This finding illuminates the importance of maintaining the close proximity of clinical team members. It also provides additional evidence that a provider's work environment including face-to-face interactions influences their intention to remain in their current positions, which is pivotal to workforce retention. This finding is aligned with previous studies that have concluded that a clinician's work environment and practice atmosphere play a significant role in burnout rates [37]. Our findings suggest that increased in-person interactions may be one isolated work environment factor that mitigates job dissatisfaction. Given the rapid and expanded use of electronic health records, it is important for policymakers, administrators, and clinicians to evaluate the effectiveness of integrated HIT systems for not only patient outcomes but also provider outcomes.

Our study also found a significant difference in smart phone use between providers based on their geographic practice location. PCPs in rural communities were less likely to use a smart phone. Emerging evidence has also noted significant perceived burdens of EHR use in rural providers [38]. As more and more EHR- and smartphone-based applications are being developed for patients and providers, it is important to specifically target rural populations to understand their accessibility, willingness, and acceptability of use of mobile and electronic health applications.

While health care organizations continue to implement team communication policies, such as those adopted in EHR systems, more research is needed to understand the varying impacts on provider outcomes [39]. Researchers have already identified some perceived burdens of EHR use reported by providers [40, 41]. It appears critical to further understand why certain aspects of electronic communication are burdensome to clinicians in primary care. Novel electronic applications that involve a user-centered design are recommended in future research. Particularly in this recent era of the global pandemic of COVID-19, where we have seen dramatic changes in the delivery of healthcare and the rise of digital approaches to care team communication and patient management, our data highlights the potential unique benefits of retaining aspects of the more traditional intimate/close proximity communication styles in the context of patient care. Particularly, in the past decade, the increase of smart phone applications and communication between providers via social media platforms has emerged. In early studies, researchers have illuminated concerns surrounding patient confidentiality and the lack of trustworthiness of content shared via social media [42, 43]. However, in one recent qualitative study about the experiences of clinicians during the early waves of the COVID-19 pandemic, researchers highlighted the beneficial use of social media for timely information sharing about patient treatment protocols, workforce safety measures, and public health policy implementation [44]. Clinician participants in another qualitative study specifically noted provider reliance on social media for the rapid dissemination of pre-published evidence needed to shift care delivery protocols and their subsequent response to the pandemic emergency [45]. More empirical and qualitative evidence about the accuracy of information, the effectiveness of social media communication on patient and provider outcomes, and ethical concerns surrounding smart phone applications and social media during provider communication is needed. Potential implications of such research may include the development of workforce policies surrounding social media use among healthcare teams, as well as leveraging the rapid and wide exchange of information to benefit a wider and hard to reach population. Coupling our data with general public health and occupational guidance to ensure the safety of providers and patients, future work exploring what key aspects of communication modalities may provide important insight into the most effective approaches to team-based care communication models is needed.

There are limitations to our study. The survey was conducted in one U.S. state, and our PCP respondents may have different responses compared to nonrespondents. However, we purposively selected New York as a state representative of the same NP and PA scope of practice policies as a large majority of other US states. In addition, the demographics of our sample were comparable to other national PCP surveys. We also recognize the variability of work environment characteristics across primary care practices that may influence provider burnout, satisfaction, and the intention to leave their current position. Yet, we made an effort to control for such confounders to capture PCP perspectives at the provider-level and focused on the specific use of communication as one of many variables that influence a provider's job satisfaction. Additional research specific to varying work environment factors is needed to further support PCP satisfaction.

## 5. Conclusions

Effective communication is a critical element for interdisciplinary team cohesiveness when two or more providers are comanaging the same primary care patient. This study provides novel evidence that in-person communication significantly reduces job dissatisfaction and the intention to leave the current position in PCPs. We also found evidence of significant differences in electronic health record and smartphone use in rural providers compared to providers in urban and suburban locations, suggesting that geographic settings influence communication infrastructure use. More research is needed to examine communication modalities that best support provider satisfaction and thus mitigate burnout.

## Figures and Tables

**Table 1:** Sample demographics and practice characteristics by location.

	Urban (*n* = 110)	Rural (*n* = 70)	Suburban (*n* = 134)	Overall (*n* = 314)	*p*
Median age (IQR^†^)	55 (46, 63)	53 (43, 61)	55 (43, 62)	55 (44, 62)	0.64

Median years of experience (IQR^†^))	20 (12, 27)	19 (10, 29)	22 (13, 31)	20 (12, 29)	0.40

Discipline *n* (%)					0.96
Nurse practitioner	51 (46.4%)	36 (51.4%)	63 (47.0%)	150 (47.8%)	
Physician	31 (28.2%)	19 (27.1%)	39 (29.1%)	89 (28.3%)	
Physician assistant	28 (25.5%)	15 (21.4%)	32 (23.9%)	75 (23.9%)	

White	80 (72.7%)	67 (97.1%)	123 (91.8%)	270 (86.3%)	**<0.001**

Hispanic	5 (4.6%)	0 (0%)	6 (4.5%)	11 (3.5%)	0.18

Female	76 (69.7%)	52 (74.3%)	109 (81.3%)	237 (75.7%)	0.10

Office setting					**0.002**
Provider-owned practice	47 (42.7%)	26 (37.1%)	84 (62.7%)	157 (50.0%)	
University/hospital affiliated clinic	45 (40.9%)	34 (48.6%)	34 (25.4%)	113 (36.0%)	
Others	18 (16.4%)	10 (14.3%)	16 (11.9%)	44 (14.0%)	

Length of time employed					0.22
3 or fewer years	18 (16.4%)	14 (20.0%)	13 (9.8%)	45 (14.4%)	
4–9 years	29 (26.4%)	16 (22.9%)	44 (33.1%)	89 (28.4%)	
10 or more years	63 (57.3%)	40 (57.1%)	76 (57.1%)	179 (57.2%)	

Type of work					**0.01**
Part-time	55 (50.0%)	37 (52.9%)	91 (67.9%)	183 (58.3%)	
Full-time	55 (50.0%)	33 (47.1%)	43 (32.1%)	131 (41.7%)	

Primary practice					**0.005**
Independent panel	41 (37.3%)	39 (55.7%)	43 (32.6%)	123 (39.4%)	
Comanaging panel	69 (62.7%)	31 (44.3%)	89 (67.4%)	189 (60.6%)	

IQR^†^: interquartile range.

**Table 2:** Frequency of smartphone use and different modes of communication by location.

	Urban (*n* = 110)	Rural (*n* = 70)	Suburban (*n* = 134)	Overall (*n* = 314)	*p*
Smartphone use *n* (%)	87 (79.1%)	40 (58.0%)	98 (73.1%)	225 (71.9%)	0.008
Provider communication	65 (74.7%)	25 (62.5%)	65 (67.0%)	155 (69.2%)	0.32
Clinical decision apps	67 (77.0%)	35 (87.5%)	78 (80.4%)	180 (80.4%)	0.38
Review test results	26 (29.9%)	8 (20.0%)	19 (19.6%)	53 (23.7%)	0.22
Search engines	65 (74.7%)	22 (55.0%)	69 (71.1%)	156 (69.6%)	0.07
Electronical prescribing	4 (4.6%)	3 (7.5%)	5 (5.2%)	12 (5.4%)	0.80

Mode of communication^†^ (frequently)					
In-person	64 (59.8%)	38 (54.3%)	95 (73.1%)	197 (64.2%)	**0.04**
Electronic health record	57 (52.8%)	28 (41.2%)	64 (49.2%)	149 (48.7%)	0.29
Telephone call	37 (34.3%)	9 (13.2%)	34 (26.6%)	80 (26.3%)	**0.02**
Text message	15 (14.9%)	0 (0%)	12 (9.7%)	27 (9.3%)	**0.005**
E-mail	15 (15.0%)	3 (4.6%)	13 (10.6%)	31 (10.8%)	**0.01**
Smartphone application	6 (6.2%)	1 (1.6%)	5 (4.2%)	12 (4.3%)	0.57

^†^ Frequency of different modes of communication was measured as frequently, often, rarely, and never. Frequency of using frequently was displayed in the table.

**Table 3:** Correlation between team or provider outcomes and mode of communication.

	In-person		Electronic health record		Telephone call		Text message		E-mail		Smartphone application	
	Statistic	*p*	Statistic	*p*	Statistic	*p*	Statistic	*p*	Statistic	*p*	Statistic	*p*
PCMI^†^	0.29	**<0.001** ^*∗∗∗*^	0.09	0.13	0.15	**0.008** ^*∗∗*^	0.15	**0.009** ^*∗∗*^	0.01	0.84	−0.06	0.29
Self-reported burnout^‡^	—	0.16	0.91	0.82	—	0.75	0.14	0.99	2.04	0.56	—	0.83
Satisfied with job^‡^	—	**0.02** ^*∗*^	5.30	0.15	—	0.63	5.51	0.14	0.57	0.90	—	0.84
Intention to leave^‡^	—	**0.02** ^*∗*^	—	0.46	—	0.86	0.76	0.86	1.29	0.73	—	0.87

Notes: frequency of different modes of communication was measured as frequently, often, rarely, and never. ^†^ Spearman's correlation coefficients were shown. ^‡^ Chi-squared tests or fisher's exact tests were used. Chi-squared values were shown. No statistics for fisher's exact tests. ^*∗*^*p* < 0.05, ^*∗∗*^*p* < 0.01, and ^*∗∗∗*^*p* < 0.001.

**Table 4:** Association of communication mode with provider burnout-related outcomes.

Outcomes	Odds ratio ^†^
In-person	
PCMI^‡^	2.53 (1.7, 3.86)^*∗∗∗*^
Self-reported burnout	0.64 (0.43, 0.92)^*∗*^
Satisfied with job	1.51 (1.05, 2.19)^*∗*^
Intention to leave	0.55 (0.36, 0.85)^*∗∗*^

Electronic health record	
PCMI^‡^	1.23 (0.92, 1.66)
Self-reported burnout	0.9 (0.67, 1.23)
Satisfied with job	1.05 (0.78, 1.4)
Intention to leave	0.87 (0.61, 1.24)

Telephone call	
PCMI^‡^	1.41 (1.03, 1.93)^*∗*^
Self-reported burnout	0.81 (0.59, 1.11)
Satisfied with job	0.92 (0.67, 1.25)
Intention to leave	1.27 (0.87, 1.88)

Text message	
PCMI^‡^	1.38 (1.03, 1.86)^*∗*^
Self-reported burnout	0.88 (0.65, 1.19)
Satisfied with job	1.21 (0.9, 1.64)
Intention to leave	1 (0.69, 1.43)

E-mail	
PCMI^‡^	1.16 (0.88, 1.55)
Self-reported burnout	0.84 (0.62, 1.12)
Satisfied with job	1.04 (0.78, 1.38)
Intention to leave	0.88 (0.62, 1.24)

Smartphone application	
PCMI^‡^	0.97 (0.68, 1.4)
Self-reported burnout	0.8 (0.53, 1.15)
Satisfied with job	1 (0.71, 1.45)
Intention to leave	1.04 (0.65, 1.59)

Notes: complete cases were analyzed in separate models. ^†^ Adjusted for occupation, age, race, gender, years of experience, office setting, length of work experience, work type, and location. ^‡^ Provider comanagement index score was dichotomized as a binary variable using the mean score 70. ^*∗*^*p* < 0.05, ^*∗∗*^*p* < 0.01, and ^*∗∗∗*^*p* < 0.001.

**Table 5:** Frequency of smartphone use and modes of communication by discipline.

	Physician (*n* = 96)	Nurse practitioner (*n* = 158)	Physician assistant (*n* = 79)	Overall (*n* = 333)	*p*
Smartphone use *n* (%)	72 (75.8%)	111 (71.2%)	55 (69.6%)	238 (72.1%)	0.62
Provider communication	47 (65.3%)	78 (70.9%)	41 (74.5%)	166 (70.0%)	0.51
Clinical decision apps	58 (80.6%)	85 (77.3%)	47 (85.5%)	190 (80.2%)	0.46
Review test results	23 (31.9%)	24 (21.8%)	11 (20.0%)	58 (24.5%)	0.20
Search engines	54 (75.0%)	79 (71.8%)	34 (61.8%)	167 (70.5%)	0.25
Electronical prescribing	2 (2.8%)	7 (6.4%)	3 (5.5%)	12 (5.1%)	0.64

Mode of communication^†^ (frequently)					
In-person	55 (58.5%)	106 (68.8%)	46 (60.5%)	207 (63.9%)	0.22
Electronic health record	45 (47.4%)	78 (51.7%)	35 (46.1%)	158 (49.1%)	0.94
Telephone call	29 (31.5%)	37 (24.7%)	20 (25.3%)	86 (26.8%)	0.51
Text message	7 (7.7%)	16 (11.3%)	9 (12.0%)	32 (10.4%)	0.93
E-mail	6 (6.6%)	22 (15.9%)	5 (6.7%)	33 (10.9%)	0.08
Smartphone application	4 (4.7%)	7 (5.3%)	3 (4.1%)	14 (4.8%)	0.60

Notes: ^†^ frequency of different modes of communication was measured as frequently, often, rarely, and never. Frequency of using frequently was displayed in the table.

## Data Availability

All data will be made available upon reasonable request.
